# Lower circulating endocannabinoid levels in children with autism spectrum disorder

**DOI:** 10.1186/s13229-019-0256-6

**Published:** 2019-01-30

**Authors:** Adi Aran, Maya Eylon, Moria Harel, Lola Polianski, Alina Nemirovski, Sigal Tepper, Aviad Schnapp, Hanoch Cassuto, Nadia Wattad, Joseph Tam

**Affiliations:** 1 Neuropediatric Unit, Shaare Zedek Medical Center, 12 Bayit Street, 91031 Jerusalem, Israel; 2 Obesity and Metabolism Laboratory, Institute for Drug Research, School of Pharmacy, Faculty of Medicine, The Hebrew University of Jerusalem, Jerusalem, Israel; 3 Department of Nutritional Sciences, Tel Hai Academic College, Upper Galilee, 1220800 Kiryat Shmona, Israel

**Keywords:** Autism spectrum disorder, Endocannabinoid system, Anandamide, *N-*arachidonoylethanolamine, 2-arachidonoylglycerol, Arachidonic acid, *N-*palmitoylethanolamine, *N-*oleoylethanolamine, Biomarkers, Cannabinoids

## Abstract

**Background:**

The endocannabinoid system (ECS) is a major regulator of synaptic plasticity and neuromodulation. Alterations of the ECS have been demonstrated in several animal models of autism spectrum disorder (ASD). In some of these models, activating the ECS rescued the social deficits. Evidence for dysregulations of the ECS in human ASD are emerging, but comprehensive assessments and correlations with disease characteristics have not been reported yet.

**Methods:**

Serum levels of the main endocannabinoids, *N-*arachidonoylethanolamine (AEA or anandamide) and 2-arachidonoylglycerol (2-AG), and their related endogenous compounds, arachidonic acid (AA), *N*-palmitoylethanolamine (PEA), and *N*-oleoylethanolamine (OEA), were analyzed by liquid chromatography/tandem mass spectrometry in 93 children with ASD (age = 13.1 ± 4.1, range 6–21; 79% boys) and 93 age- and gender-matched neurotypical children (age = 11.8 ± 4.3, range 5.5–21; 79% boys). Results were associated with gender and use of medications, and were correlated with age, BMI, and adaptive functioning of ASD participants as reflected by scores of Autism Diagnostic Observation Schedule (ADOS-2), Vineland Adaptive Behavior Scale-II (VABS-II), and Social Responsiveness Scale-II (SRS-2).

**Results:**

Children with ASD had lower levels (pmol/mL, mean ± SEM) of AEA (0.722 ± 0.045 vs. 1.252 ± 0.072, *P* < 0.0001, effect size 0.91), OEA (17.3 ± 0.80 vs. 27.8 ± 1.44, *P* < 0.0001, effect size 0.94), and PEA (4.93 ± 0.32 vs. 7.15 ± 0.37, *P* < 0.0001, effect size 0.65), but not AA and 2-AG. Serum levels of AEA, OEA, and PEA were not significantly associated or correlated with age, gender, BMI, medications, and adaptive functioning of ASD participants. In children with ASD, but not in the control group, younger age and lower BMI tended to correlate with lower AEA levels. However, these correlations were not statistically significant after a correction for multiple comparisons.

**Conclusions:**

We found lower serum levels of AEA, PEA, and OEA in children with ASD. Further studies are needed to determine whether circulating endocannabinoid levels can be used as stratification biomarkers that identify clinically significant subgroups within the autism spectrum and if they reflect lower endocannabinoid “tone” in the brain, as found in animal models of ASD.

**Electronic supplementary material:**

The online version of this article (10.1186/s13229-019-0256-6) contains supplementary material, which is available to authorized users.

## Introduction

Cannabis has a well-known effect on social behavior in humans [1]. It enhances interpersonal communication [2] and decreases hostile feelings [3]. The principle unique compounds in the cannabis plant (phytocannabinoids) are Δ^9^-tetrahydrocannabinol (THC) and cannabidiol (CBD). The main target of THC in the brain is the cannabinoid type-1 receptor (CB_1_R), which is highly expressed in the frontal cortex and subcortical areas associated with social functioning [4]. The CB_1_R and its endogenous ligands *N-*arachidonoylethanolamine (anandamide or AEA) and 2-arachidonoil-glycerol (2-AG) regulate social play and anxiety in animal models [5–7] and in humans [8, 9]. Pharmacological and genetic experiments in animal models suggest that social reward is regulated by oxytocin-dependent activation of the endocannabinoid system (ECS) in the nucleus accumbens, the primary rewarding center in the brain [10]. Moreover, the ECS is a major regulator of synaptic plasticity (excitatory and inhibitory) through long-term potentiation and long-term depression [11].

Endocannabinoids (AEA and 2-AG) are usually produced “on demand” in the post-synaptic neuron and act as retrograde signaling messengers. By activating CB_1_R in the pre-synaptic neuron, both endogenous ligands result in decreased release of neurotransmitters into the synaptic cleft and attenuate over-active brain circuits [11]. Reduced endocannabinoid “tone” was suggested to be involved in the pathogenesis of ASD in several animal models: monogenic (fragile X [12, 13], neuroligin 3 [14]), polygenic (BTBR [15]), and environmental (rat valproic acid [16, 17]). Activation of the ECS in these and other models of ASD reversed the autistic symptoms [13, 15, 17–23].

Several human studies revealed associations between polymorphisms in the gene encoding CB_1_R and emotions’ processing [24–26]. Other studies demonstrated reduced expression of CB_1_R in postmortem brains of individuals with autism [27] as well as reduced plasma AEA levels in children with ASD [28]. However, these studies were not designed to comprehensively characterize the involvement of the ECS in the pathogenesis of ASD. Therefore, we assessed the circulating levels of several endocannabinoids and delineate the correlations between their levels and disease characteristics in a large group of children with ASD and their matched controls with typical development.

## Methods

### Participants

One hundred and eighty-six children and young adults, age 5.5 to 21 years, participated in this study. Ninety-three participants with ASD had been recruited as part of an ongoing randomized clinical trial (NCT02956226) that assesses the safety, tolerability, and efficacy of two CBD compounds in 150 participants with ASD and behavioral problems. Legal guardians of 93 participants consented to the assessment of serum endocannabinoids at baseline. Ninety-three unrelated, age- and gender-matched, control participants, attending or graduating regular education and without any neuropsychiatric diagnosis other than ADHD, were recruited through advertisements posted in the surrounding community. Participant characteristics are presented in Table 1.

**Table 1:** Participant characteristics

	Neurotypical control	Children with ASD
*N*	93	93
Age	11.8 ± 4.3	13.1 ± 4.1^
% male	79%	79%
BMI	21.0 ± 4.2	20.4 ± 5.5
Epilepsy comorbidity	0%	10%
High ASD symptoms severity		
ADOS comparison score = 8–10		77%
VABS standard score ≤ 70		88%
CARS total score ≥ 37		81%
SRS *t* scores ≥ 75		86%
Psychotropic medications*		
Any		80%
Antipsychotic		56%
SSRIs		23%
Stimulants		15%
Antiepileptic (mood stabilizers)		13%
Benzodiazepines		8%
Others		5%

**^**Significant age difference (*P* = 0.040). The BMI difference was not significant

*Medications were stable for at least 1 month before blood collection. *Antipsychotic* (*n*): risperidone (21), aripiprazole (16), clotiapine (10), periciazine (10), olanzapine (5), promethazine (3), quetiapine fumarate (seroquel, 3), methotrimeprazine (1). *SSRIs* selective serotonin reuptake inhibitors (*n*): fluoxetine (15), fluvoxamine (2), sertraline (2), escitalopram (1), trazodone (1). *Stimulants* (*n*): methylphenidate (11), lisdexamfetamine (2). *Antiepileptic* (*n*): valproic acid (7), carbamazepine (1), lamotrigine (1), topiramate (1), stiripentol (1), sulthiame (1). *Benzodiazepines* (*n*): clobazam (3), clonazepam (3), zolpidem (1). Others (*n*): guanfacine (2), propranolol (2), enalapril (1)

### Behavioral assessments

Behavioral assessments included a confirmation of ASD diagnosis based on *Diagnostic and Statistical Manual of Mental Disorders, Fifth Edition* (DSM-5) [29] criteria and the autism diagnostic observation schedule second edition (ADOS-2) [30] and the following assessments:

Vineland Adaptive Behavior Scale-II (VABS-II) [31]. The VABS is a semi-structured caregiver interview designed to assess functional skills in five domains, each with two to three subdomains. The main domains are communication, daily living skills, socialization, motor skills, and maladaptive behavior.

*Social Communication Questionnaire (SCQ) Lifetime Form* [32]. This is a 40-item, parent-report screening measure*.* The SCQ total score comprises items assessing reciprocal social interaction, communication, and restricted, repetitive, and stereotyped patterns of behavior.

*Childhood Autism Rating Scale-second edition (CARS2-ST)* [33]. This is a quantitative measure of direct behavior observation consists of 15 scales that cover various aspects of interactive behavior—communication, body use, the child’s response to stimuli, and activity level.

*Home Situations Questionnaire-*Autism *Spectrum Disorder (HSQ-ASD)* [34]. This is a 24-item parent-rated measure of non-compliant behavior in children with ASD. The HSQ-ASD was translated into Hebrew with the permission of the authors using a translation and retranslation by two bilingual professionals and validated for this study.

*Child Behavior Checklist (CBCL-validated Hebrew version)* [35, 36]. A screening tool of emotional and behavioral problems in 4- to 18-year-old children. The CBCL generates three broadband results—an internalizing score, an externalizing score (summing up non-compliant scale and aggressive behavior scale), and a total score. For this study, we used the externalizing section only.

*Autism Parenting Stress Index (APSI)* [37]*.* This is a 13-item parent-rated measure designed to assess the effect of interventions to control disruptive behavior in children with ASD on parenting stress.

*Social Responsiveness Scale-II (SRS-2, Hebrew version)* [38, 39]. This is a 69–71 item, caregiver (pSRS) or teacher (tSRS) questionnaire, used to determine the severity of social deficit exhibited by participants with ASD. The SRS contains five subscales: social awareness, social cognition, social communication, social motivation, and autistic mannerisms, which respectively measure the ability to recognize social cues, the ability to interpret social cues, the ability to use expressive verbal and non-verbal language skills, the ability to engage in social-interpersonal behaviors, and the tendency to display stereotypical behaviors and restricted interests characteristic of autism.

*Clinical Global Impression-Severity (CGI-S)* [40]. This is a 7-point scale designed to measure severity of illness (CGI-S) by trained clinicians. Scores in the severity scale range from 1 (normal) through to 7 (amongst the most severely ill patients).

### Sample preparation and endocannabinoids measurements

Blood collection was performed between 10 am and 2 pm. Whole blood samples were collected into 5 mL VACUETTE® Serum Clot Activator Tubes (VWR, PA, USA), and after 30 min were centrifuged (3000×*g* at 4 °C for 10 min). The serum fraction was aliquoted into polypropylene tubes and immediately stored at − 80 °C.

The extraction, purification, and quantification of serum endocannabinoids were performed by stable isotope dilution liquid chromatography/tandem mass spectrometry (LC-MS/MS) as previously described [41]. Briefly, AEA, 2-AG, arachidonic acid (AA), *N*-palmitoylethanolamine (PEA), and *N*-oleoylethanolamine (OEA) in serum samples were extracted, purified, and quantified using the stable isotope dilution LC-MS technique. Total proteins were first precipitated using ice-cold acetone and Tris buffer (50 mM, pH 8.0). Next, samples were homogenized using a mixture of 0.5 mL ice-cold methanol/Tris buffer (50 mM, pH 8.0), 1:1, and 7 μL internal standard (22.4 ng d_4_-AEA). Following this, the homogenates were extracted using ice-cold CHCl_3_:MeOH (2:1, *v*/*v*) after they were washed with ice-cold chloroform three times. The samples were then dried under thin stream of nitrogen and reconstituted in MeOH. Analysis by LC-MS was conducted on an AB Sciex (Framingham, MA, USA) Triple Quad 5500 Mass Spectrometer and a Shimadzu (Kyoto, Japan) UHPLC System, while the liquid chromatographic separation was acquired via a Kinetex (Phenomenex) column (C18, 2.6 mm particle size, 100*2.1 mm). Sample levels of AEA, 2-AG, AA, PEA, and OEA were measured against a standard curve and then expressed as pmol/mL.

### Statistical analysis

Statistical analyses were performed using SPSS version 23 (SPSS Inc., Chicago, IL, USA) software. Continuous variables are presented as mean ± SD with the exception of the serum endocannabinoid levels that are being presented as mean ± SEM, as specified in the text. To test the differences in continuous variables between participants with ASD and neurotypical control group, the independent samples *t* test was performed, a correction for multiple comparisons was conducted using Bonferroni correction. The Mann-Whitney test was not needed based on normal/near normal distribution of OEA, PEA, and AEA. Associations between nominal variables were performed with the Pearson *χ2* test. Pearson correlation was used to test the correlation between endocannabinoids’ levels and demographic parameters or adaptive functioning of participants. A multivariate logistic regression analysis was performed to test the independent association between the serum endocannabinoid levels and ASD status, adjusting for age, gender, and BMI, which are potential confounders.

A linear regression analysis was performed to test the correlations of serum endocannabinoid levels with age, gender, and BMI. Finally, to ease clinical interpretation and sensitivity analysis, endocannabinoids were analyzed also as dichotomous variables defined by a cut-off of below the 25th percentile (first quartile) value for each endocannabinoid in the control group. *P* < 0.05 was considered statistically significant for all analyses.

## Results

### Serum endocannabinoids are lower in ASD

Serum levels of the main endocannabinoid *AEA and its structurally related compounds OEA* and *PEA* were lower in children with ASD versus age-, gender-, and BMI-matched control group of typically developed children.

**Fig. 1:**
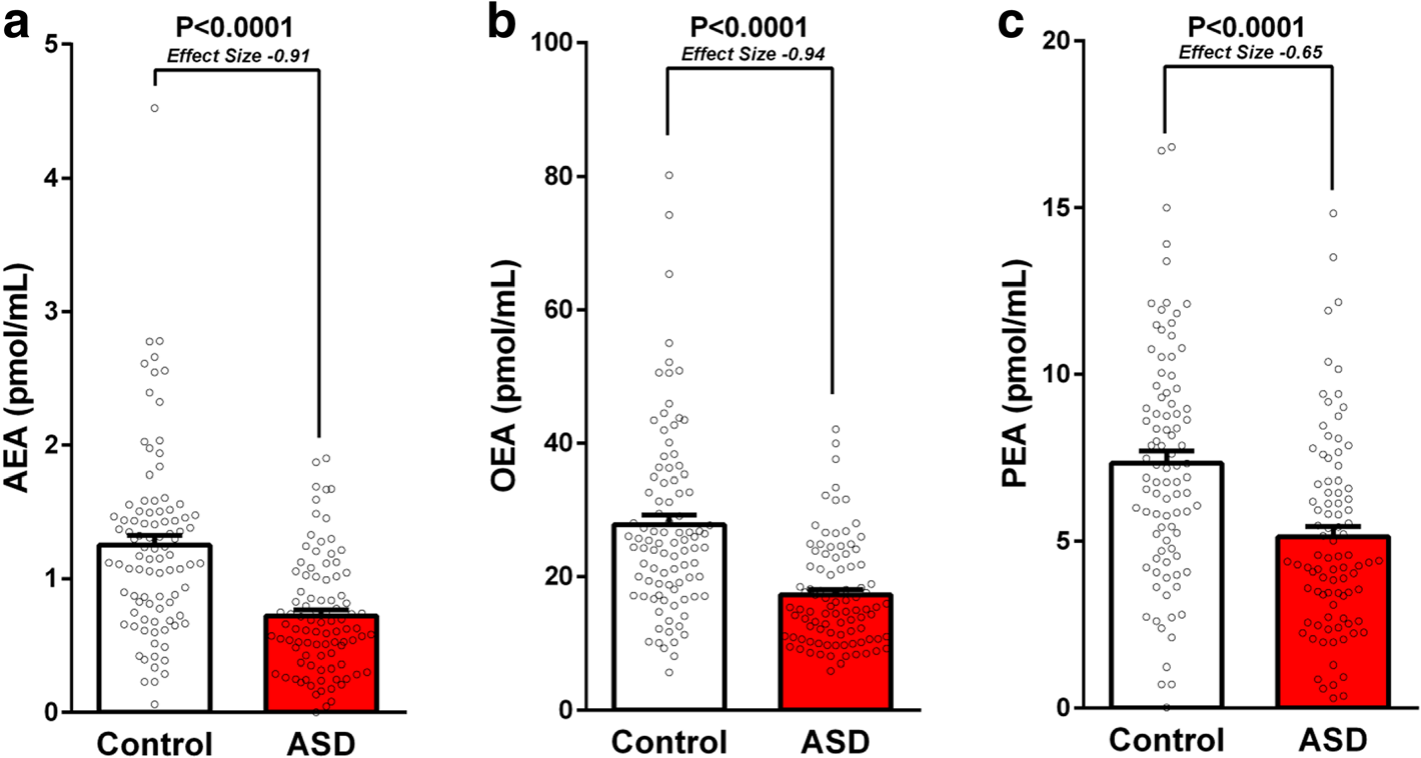
Lower serum endocannabinoid levels in children with ASD. Legend: low endocannabinoid “tone” in serum samples of 93 children with ASD compared with 93 age- and gender-matched controls. Results of anandamide (AEA; panel **a**), oleoylethanolamine (OEA; panel **b**), and palmitoylethanolamide (PEA; panel **c**) are presented as mean, standard error, and distribution respectively

[Fig. 1; *AEA* 0.722 ± 0.045 vs. 1.252 ± 0.072, *P* = 2.9E-09, Cohen’s *d* effect size; 0.91. *OEA* 17.3 ± 0.80 vs. 27.8 ± 1.44, *P* = 1.2E-09, Cohen’s *d* effect size; 0.94. *PEA* 4.93 ± 0.32 vs. 7.15 ± 0.37, *P* = 1.4E-05, Cohen’s *d* effect size; 0.65 pmol/mL, mean ± SEM, these differences remained statistically significant after a correction for multiple comparisons]. Serum levels of 2-AG and its breakdown molecule AA were not significantly different between groups [*2-AG* 29.91 ± 5.29 vs. 18.92 ± 1.89, *P* = 0.12; *AA* 2092 ± 93 vs. 2228 ± 133, *P* = 0.39; ASD vs. control, pmol/mL, mean ± SEM].

### Serum endocannabinoids differentiated ASD and controls

Serum levels of AEA, OEA, and PEA were independently associated with ASD status when adjusting for age, gender, BMI, and attention deficit hyperactivity disorder (ADHD) status. (Additional file 1: Table S1, *P* = 0.001, 0.01 and 0.007, respectively). Cut-off level for AEA, OEA, and PEA were set at the 25th percentile (first quartile) value in the control group. These values are presented in Table 2 with sensitivity and specificity in ASD detection and *P* values.

**Table 2:** Sensitivity and specificity of low serum levels (below cut-off) of AEA, OEA, and PEA

	AEA	OEA	PEA	AEA and OEA	AEA and PEA	OEA and PEA	AEA and OEA and PEA	AEA or OEA or PEA
Cut-off level^	0.76	18.5	4.6					
ASD^#^	63%	68%	56%	59%	41%	46%	40%	81%
Control^#^	25%	25%	25%	19%	13%	15%	12%	39%
*P* value	3.8E-8	7.9E-10	9.8E-6	7.9E-9	1.17E-5	2.3E-6	8.8E-6	1.17E-9
Sensitivity	63%	68%	56%	59%	41%	46%	40%	81%
Specificity	75%	75%	75%	81%	87%	85%	88%	61%

**^**Cut-off levels were set at the 25th percentile in the control group. Results are presented in pmol/mL

^**#**^Participants below cut-off

### Impact of age, gender, and BMI on serum endocannabinoid levels

Difference in endocannabinoid levels between genders and correlations with age and BMI appear in Table 3. Younger and thinner children with ASD had lower levels of serum AEA (Table 3). However, these correlations did not remain significant after a correction for multiple comparisons. No other significant correlations of AEA, OEA, and PEA with age and BMI or associations with gender were found.

**Table 3:** Correlations of serum endocannabinoid levels with age and BMI and association with gender^$^

	Age			Gender			BMI		
	Control	ASD	Overall	Control	ASD	Overall	Control	ASD	Overall
AEA	0.21 (0.26)	0.31 (0.002)^#^	− 0.02 (0.81)	0.13 (0.20)	0.31 (0.77)	0.08 (0.26)	0.21 (0.26)	0.31 (0.002)^#^	0.30 (0.001)^*#*^
PEA	− 0.15 (0.16)	− 0.43 (0.68)	− 0.14 (0.05)	0.23 (0.03)^#^	0.46 (0.66)	0.14 (0.06)	0.07 (0.72)	− 0.06 (0.54)	− 0.03 (0.77)
OEA	− 0.12 (0.26)	0.20 (0.06)	− 0.72 (0.33)	0.12 (0.27)	0.01 (0.91)	0.07 (0.36)	0.17 (0.38)	0.12 (0.25)	0.13 (0.14)

^$^Linear regression models; results are presented as *R* (*P* value)

^#^Results are not significant after a correction for multiple comparisons

### Impact of ADHD symptoms, ASD severity, comorbidity, and use of medications on serum endocannabinoid levels

**Fig. 2:**
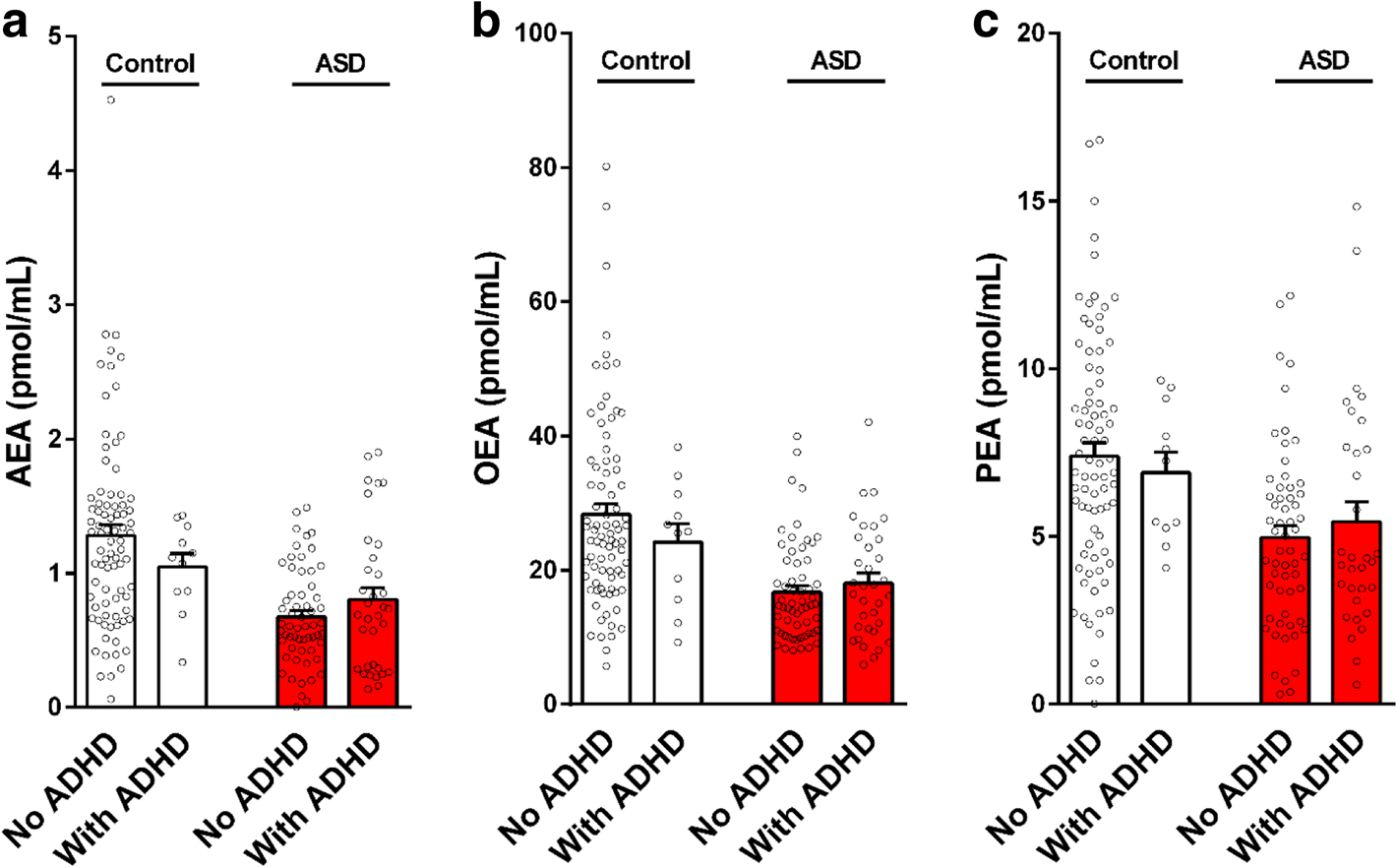
Serum endocannabinoid levels in children with ASD and matched controls stratified by ADHD symptoms. Legend: 11 out of 93 children in the control group and 34 out of 93 children in the ASD group had ADHD symptoms. In both groups, there were no significant differences between the endocannabinoid levels in children with and without ADHD symptoms. Results of AEA (panel **a**),  OEA (panel **b**) and PEA (panel **c**) are presented as mean, standard error, and distribution

**Fig. 3:**
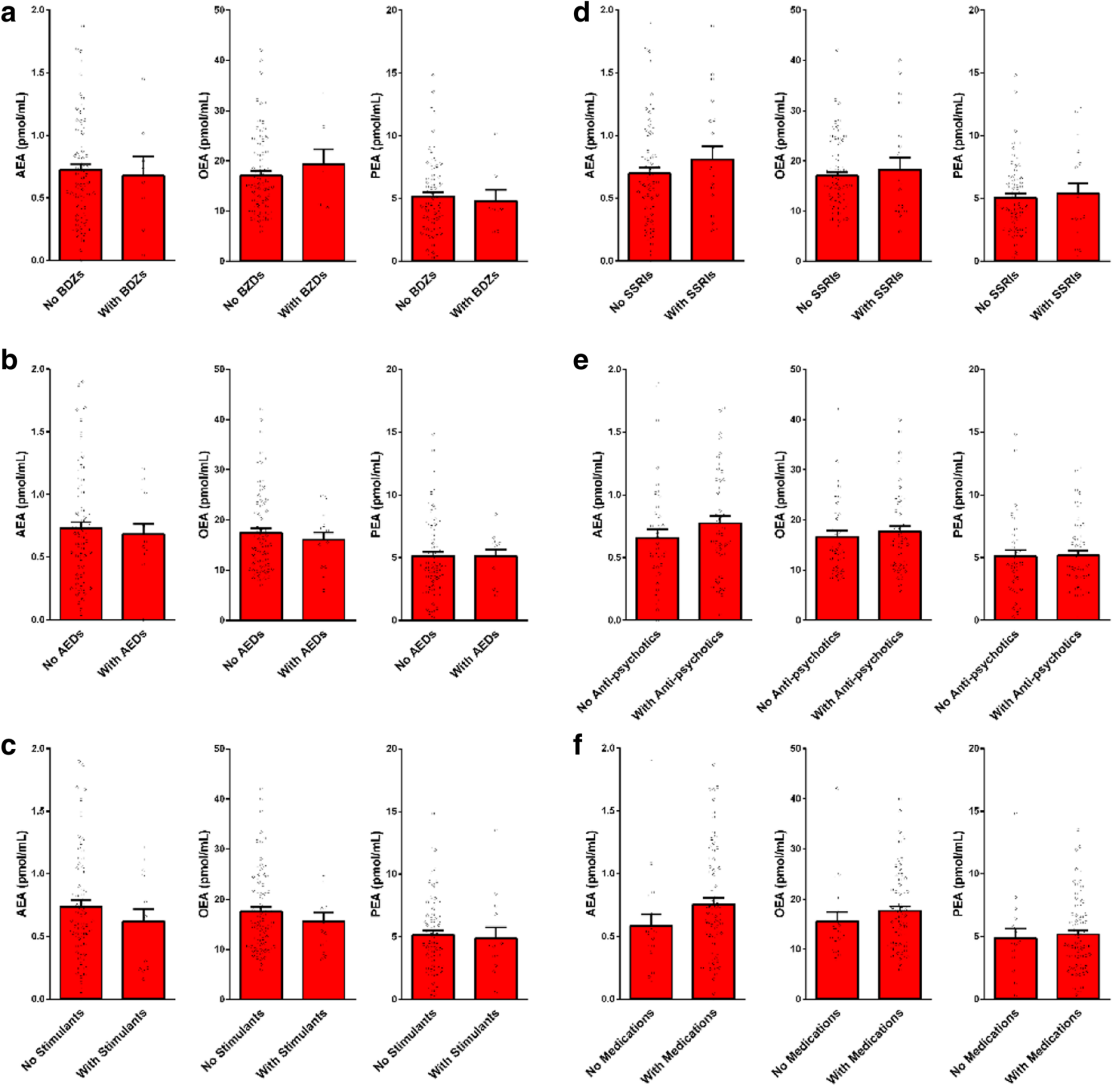
Impact of medication use on serum endocannabinoid levels in children with ASD. Legend: endocannabinoid levels in serum samples of 93 children with ASD stratified by medication use. There were no significant differences between the endocannabinoid levels in either of these subgroups. **a** Benzodiazepines (*n* = 8). **b** Antiepileptic drugs (*n* = 14). **c** Stimulants (*n* = 14). **d** Selective serotonin reuptake inhibitors (*n* = 20). **e** Anti-psychotics (*n* = 41). **f** Any medication (*n* = 74). Results are presented as mean, standard error, and distribution

**Fig. 4:**
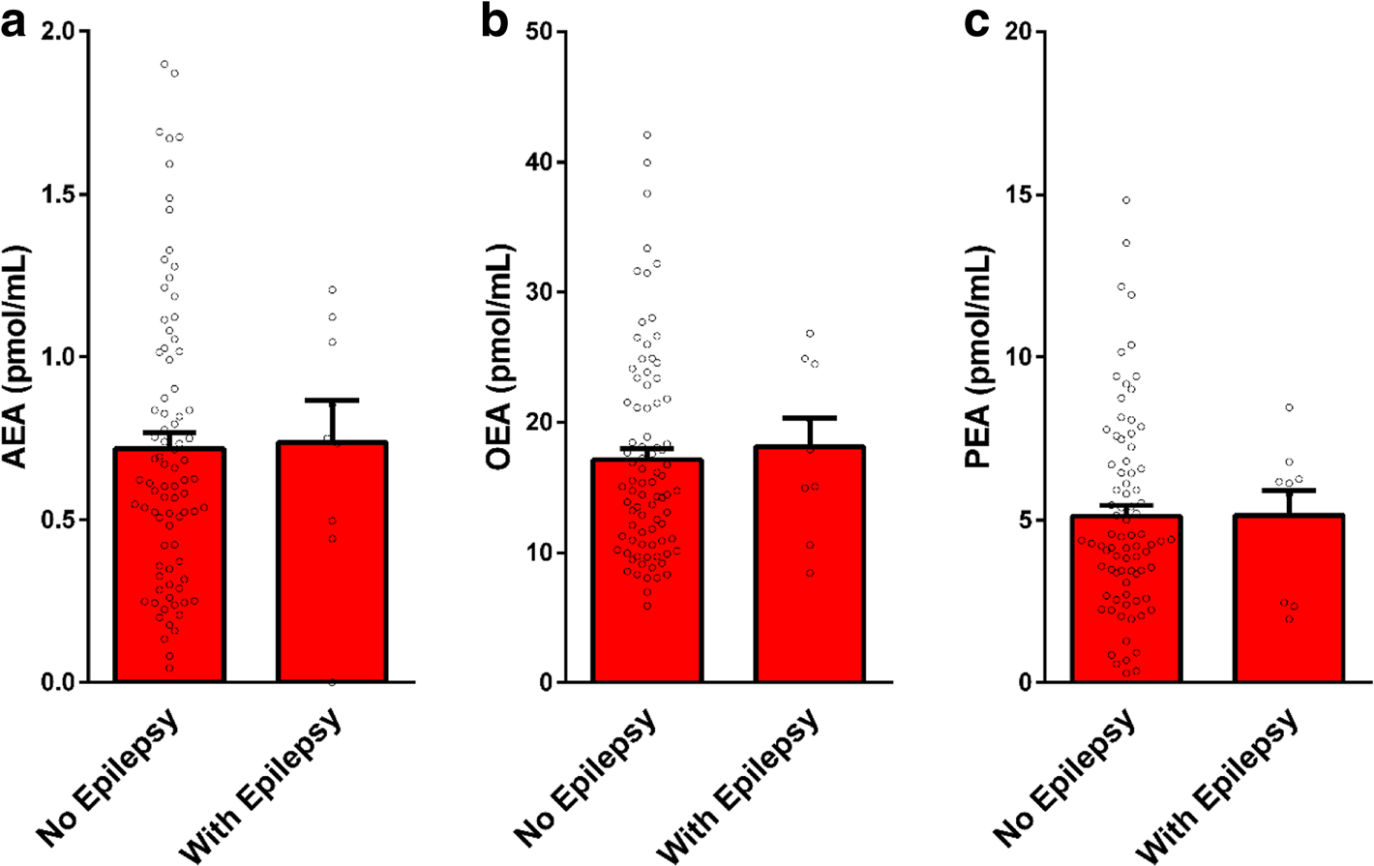
Impact of epilepsy status on serum endocannabinoid levels in children with ASD. Legend: endocannabinoid levels in serum samples of 93 children with ASD—with (*n* = 9) and without (*n* = 84) epilepsy. There were no significant differences between children with and without epilepsy. Results of AEA (panel **a**), OEA (panel **b**) and PEA (panel **c**)  are presented as mean, standard error, and distribution

Correlations of circulating AEA, OEA, and PEA levels with adaptive functioning and associations with ADHD, epilepsy comorbidity, use of medications and history of prenatal complications are presented in Table 4 and Figs. 2, 3, and 4. No significant correlations or associations were found. Some tendency toward a correlation was observed for AEA with APSI (*R* = 0.19, *P* = 0.06) and with use of antipsychotic medications (*R* = 0.18, *P* = 0.07). Children with higher APSI severity score or with higher use of antipsychotics had lower AEA levels.

**Table 4:** Impact of ASD participants’ characteristics on serum endocannabinoid levels

	AEA	OEA	PEA
ADOS comparison score [*R* (*P*)]^#^	0.01 (0.9)	0.06 (0.5)	0.07 (0.4)
VABS standard score [*R* (*P*)]^#^	− 0.02 (0.8)	− 0.05 (0.6)	0.09 (0.4)
CARS summary score [*R* (*P*)]^#^	− 0.15 (0.15)	− 0.08 (0.3)	− 0.03 (0.7)
DSM-5: level of support required in social communication [*R* (*P*)]^#^	− 0.11 (0.29)	0.01 (0.89)	0.03 (0.9)
DSM-5: level of support required in repetitive restricted behavior [*R* (*P*)]^#^	− 0.01 (0.9)	0.01 (0.89)	− 0.01 (0.7)
CGI-S, behavior [*R* (*P*)]^#^	− 0.09 (0.3)	0.05 (0.2)	0.13 (0.2)
CGI-S, anxiety [*R* (*P*)]^#^	0.08 (0.8)	0.11 (0.6)	0.07 (0.4)
Family history of ASD [yes/no; *χ2* (*P*)]^#^	1.13 (0.6)	0.11 (0.73)	1.05 (0.58)
Perinatal complications [yes/no; *χ2* (*P*)]^#^	0.02 (0.8)	0.53 (0.4)	0.44 (0.8)
Epilepsy comorbidity [yes/no; *χ2* (*P*)]^#^	0.26 (0.6)	0.64 (0.42)	2.18 (0.3)
ADHD [yes/no; *χ2* (*P*)]^#^	1.84 (0.07)	1.12 (0.26)	1.5 (0.13)
Medications			
Any [yes/no; *χ2* (*P*)]^#^	1.71 (0.19)	1.45 (0.2)	3.51 (0.17)
Antipsychotic [number; *R* (*P*)]^#^	0.18 (0.07)	0.14 (0.17)	0.02 (0.8)
SSRIs [yes/no; *χ2* (*P*)]^#^	0.13 (0.7)	0.68 (0.7)	0.48 (0.7)
Stimulants [yes/no; *χ2* (*P*)]^#^	0.93 (0.6)	0.94 (0.3)	2.10 (0.3)
Antiepileptic [yes/no; *χ2* (*P*)]^#^	0.45 (0.5)	0.58 (0.45)	0.81 (0.37)
HSQ total score [*R* (*P*)]^#^	0.07 (0.5)	− 0.04 (0.7)	0.03 (0.8)
APSI—total score [*R* (*P*)]^#^	− 0.19 (0.06)	− 0.03 (0.7)	0.08 (0.4)
Parents SRS—*t* score [*R* (*P*)]^#^	− 0.48 (0.6)	0.06 (0.3)	− 0.08 (0.4)
CBCL—externalizing score [*R* (*P*)]^#^	0.04 (0.6)	0.12 (0.2)	0.08 (0.4)
SCQ—summary score [*R* (*P*)]^#^	− 0.02 (0.8)	0.05 (0.6)	0.01 (0.8)

^#^Results are presented as Pearson correlation coefficient *R*, (*P* value) for continuous variables and as Pearson chi-square *χ2* (*P* value) for dichotomous variables (AEA, OEA, PEA cut-off levels)

## Discussion

**Fig. 5:**
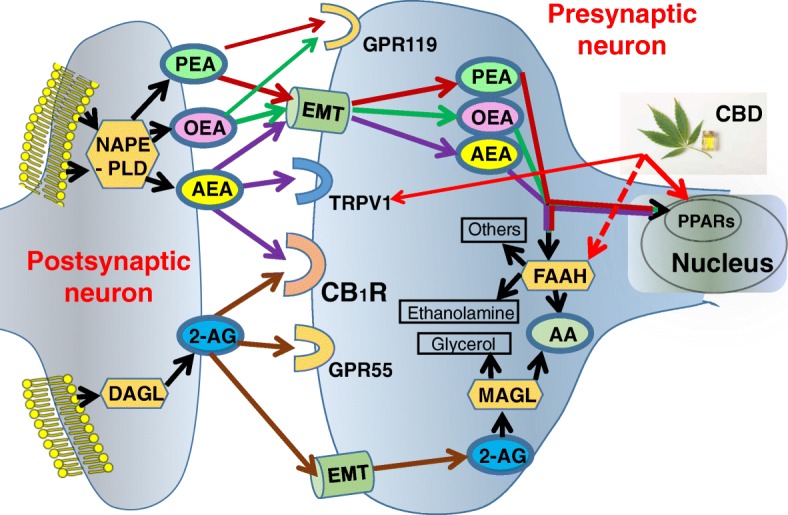
Schematic diagram of the endocannabinoid system, relevant to this study. Legend: biosynthesis, degradation, and receptors’ binding of AEA, 2-AG, OEA, and PEA are presented. AEA, PEA, and OEA are synthesized from the membrane’s phospholipids by *N*-acylphosphatidylethanolamine-specific phospholipase D (NAPE-PLD). PEA and OEA do not bind *CB*_*1*_*R*, but they can enhance *AEA* activity at transient receptor potential channels of vanilloid type-1 (TRPV1). AEA, PEA, and OEA are all degraded by fatty acid amide hydrolase (FAAH) and hence OEA and PEA can increase AEA levels by competing with AEA for FAAH (mainly OEA) or by downregulating FAAH expression (mainly PEA). Cannabidiol (CBD), a non-psychoactive component of the cannabis plant, activates peroxisome proliferator-activated receptors (PPARs) and TPRV1 and inhibits FAAH and hence might compensate for lower levels of AEA, OEA, and PEA in children with ASD. DAGL, diacylglycerol lipase; MAGL, monoacylglycerol lipase. EMT, endocannabinoid membrane transporter; GPR55, G protein-coupled receptor 55

This study provides evidence that serum levels of certain endocannabinoids are substantially decreased in people with ASD across age groups, adaptive functioning, BMIs, and in both genders. The most studied endogenous ligands of CB_1_R and cannabinoid type-2 receptor (CB_2_R) are AEA and 2-AG. We detected decreased serum concentrations of AEA and its structurally related compounds OEA and PEA with no significant differences in serum 2-AG and its breakdown molecule, AA. OEA and PEA are widely distributed in the CNS, but their classification as endocannabinoids is debatable, given their lack of affinity for CB_1_R and CB_2_R. Similar to AEA, they are, however, agonists for PPARα and GPR119 (Fig. 5).

Endocannabinoids are not stored in any cellular compartment for later use. They are generated on demand at the post-synaptic neuron cell membrane and are rapidly inactivated by pre-synaptic neuron cellular uptake and enzymatic hydrolysis (Fig. 5). As a result, the concentrations of the various endocannabinoids in the brain are constantly regulated, and even small changes might be clinically meaningful and reveal brain pathology.

Circulating endocannabinoids are believed to come from multiple organs and tissues, including the brain, skeletal muscle, adipose tissue, liver, and kidney as well as circulating cells [42]. However, plasma endocannabinoid levels were demonstrated to reflect brain concentrations [43], and hence these biomarkers are not only accessible, easy to measure, and cost-effective but can also shed a light on the pathophysiology of CNS disorders. Indeed, dysregulation of the ECS was demonstrated in many different models of ASD [44] and some of these studies convincingly illuminate previously unknown pathophysiological mechanisms. For example, prenatal exposure of rats to valproic acid is known to induce selective deficits in social play behavior and stereotypies in the offspring. In a recent study, prenatal exposure of rats to valproic acid was demonstrated to alter phosphorylation of CB_1_R, and enhancing AEA signaling through inhibition of its degradation reversed the behavioral deficits displayed by valproic acid-exposed animals [17].

Despite extensive evidence of the involvement of the ECS in animal models of ASD, human studies are sparse. Recently, Karhson and colleagues demonstrated lower levels of AEA in stored plasma samples of 59 young children (aged 3–12 years) with ASD and 53 neurotypical children (*P* = 0.034). This study was not powered to determine the relationship between plasma AEA concentrations and ASD symptom severity. The current study corroborates their finding of lower circulating AEA levels in ASD in a larger cohort and older age group, and indicates that substantial difference exists also in the levels of the AEA-like molecules PEA and OEA, but not in the levels of the other principle endocannabinoid, 2-AG. Future studies should explore possible explanations for these findings such as reduced activity of *N*-acyl phosphatidylethanolamine-specific phospholipase D (NAPE-PLD), the enzyme that synthesizes these 3 endocannabinoids, as was suggested by Siniscalco et al. [45] or increased activity of fatty acid amide hydrolase (FAAH), the enzyme that degrades them.

Notably, dysregulation of the ECS have been also implicated in the pathogenesis of epilepsy [46–48], leading eventually to phase 3 clinical studies of CBD, a non-psychoactive phytocannabinoid, for refractory epilepsy [49–51], and the recent approval of CBD by the US Food and Drug Administration to treat severe forms of epilepsy (Lennox-Gastaut syndrome and Dravet syndrome) (https://www.fda.gov/NewsEvents/Newsroom/PressAnnouncements/ucm611046.htm). These findings are of specific importance for people with ASD, as 10–30% of people with ASD have comorbid epilepsy [52], and several synaptic plasticity pathways appear to be involved in both disease processes [11]. Moreover, our findings point to a theoretical mechanism by which CBD might compensate for ECS dysregulation in ASD.

Similar to OEA and PEA, CBD has a very low affinity to CB_1_R and CB_2_R and like AEA, OEA, and PEA it may exert its CNS effects by activation of PPARs and TRPV1 [53, 54]. CBD also inhibits the enzyme FAAH [54, 55] and leads to increased levels of AEA, OEA, and PEA (Fig. 5). Therefore, our study proposes a theoretical pathophysiological mechanism for CBD in ASD and support the rationale in the ongoing and emerging clinical trials of CBD in ASD (NCT03537950, NCT02956226, NCT03202303).

Our findings also suggest the use of circulating endocannabinoids as biomarkers for ASD. In recent years, research efforts on neurological disorders are focused on identifying biomarkers to aid in diagnosis, provide prognostic information, and monitor treatment response. This is specifically imperative in ASD due to the high complexity and heterogeneity of the disorder and the importance of early diagnosis and early behavioral intervention to improve outcome. We found that circulating AEA, OEA, and PEA are substantially lower in children with ASD with a relatively high specificity and sensitivity. ASD includes a range of neurodevelopmental disorders that are very heterogeneous both clinically and etiologically, and it is not realistic to look for a specific biomarker that perfectly classify ASD and neurotypical participants. However, circulating AEA, OEA, and PEA might be used to identify a biologically homogeneous subgroup of ASD, predict response to treatments and adverse reactions to medications, and assist in the development of novel drugs that target specific core symptoms of ASD. It can also be used as a part of a multidimensional biomarker panel to predict autism risk prior to the onset of behavioral abnormalities, initiate early interventions, and predict the developmental trajectory of children with ASD. We found that AEA levels were lower in the younger children with ASD in our cohort (inverse correlation with age). As circulating endocannabinoids can be assessed in infants, future studies that evaluate their levels in infants at risk for developing ASD (e.g., siblings of children with ASD) are recommended. Future studies should also assess circulating endocannabinoids in the context of other biomarkers to evaluate their potential in a multidimensional biomarker panel,

Finally, this study has several limitations. The age range of the cohort and the adaptive level of the ASD participants were relatively wide. We could not completely control the effects of ASD comorbidities and the medications’ effect as most of our ASD participants received medications, and some medications could impact the levels of circulating endocannabinoids. In addition, we did not assess genetic, electrophysiological, and imaging biomarkers.

## Conclusions

We found lower levels of the endocannabinoids AEA, OEA, and PEA in serum samples of 93 children with ASD compared with samples of matched neurotypical control group. These findings are in line with the results of numerous former studies in animal models of ASD as well as an initial human study that demonstrated lower endocannabinoid tone in ASD. Our findings suggest the use of circulating AEA, OEA, and PEA as stratifying biomarkers of ASD and future studies should assess the clinical significance of this stratification. These markers can also be easily measured longitudinally in humans and in animal models alike, and future studies should evaluate their potential to assist in the monitoring of treatment response. Further studies are needed to determine whether circulating endocannabinoid levels are also lower in infants and can assist in pre-symptomatic diagnosis and if they reflect lower endocannabinoid tone in the brain, as found in animal models of ASD.

## Additional file

Additional file 1: **Table S1.** Serum levels of AEA, OEA, and PEA are independently associated with ASD status when adjusting for age, gender, BMI, and ADHD (logistic regression). (DOCX 64 kb)

## Abbreviations

2-AG: 2-Arachidonoylglycerol
ADHD: Attention deficit hyperactivity disorder
ADOS: Autism diagnostic observation schedule
AEA: *N-*arachidonoylethanolamine (Anandamide)
APSI: Autism Parenting Stress Index
ASD: Autism spectrum disorder
CARS: Childhood Autism Rating Scale
*CB*_*1*_*R, CB*_*2*_*R*: Cannabinoid receptor type-1, type-2
CBCL: Child Behavior Checklist
*CGI-S*: Clinical global impression-severity
DAGL: Diacylglycerol lipase
DSM-5: Diagnostic and Statistical Manual of Mental Disorders, Fifth Edition
EMT: Endocannabinoid membrane transporter
FAAH: Fatty acid amide hydrolase
GPR55: G protein-coupled receptor 55
HSQ-ASD: Home Situations Questionnaire-Autism Spectrum Disorder
LC-MS/MS: Liquid chromatography with tandem mass spectrometry
MAGL: Monoacylglycerol lipase
NAPE-PLD: *N*-acylphosphatidylethanolamine-specific phospholipase D
OEA: *N*-oleoylethanolamine
PEA: *N*-palmitoylethanolamide
PPARs: Peroxisome proliferator-activated receptors
SCQ: Social Communication Questionnaire
SRS-2: Social Responsiveness Scale-II
SSRI: Selective serotonin reuptake inhibitory
TRPV1: Transient receptor potential channels of vanilloid type-1
VABS: Vineland Adaptive Behavior Scale
