# Supplementary material for: Lower circulating endocannabinoid levels in children with autism spectrum disorder
Source: Mol Autism. 2019 Jan 30;10:2. doi: 10.1186/s13229-019-0256-6 (PMC6354384; doi:10.1186/s13229-019-0256-6)

Table S1: Serum levels of AEA, OEA and PEA are independently associated with ASD status when adjusting for age, gender, BMI and ADHD (Logistic regression).

**AEA**


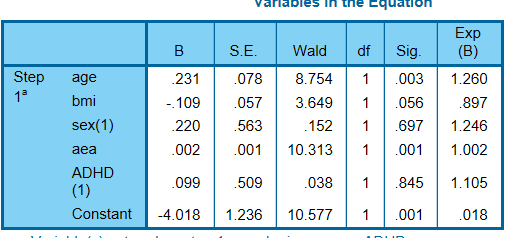


**OEA**


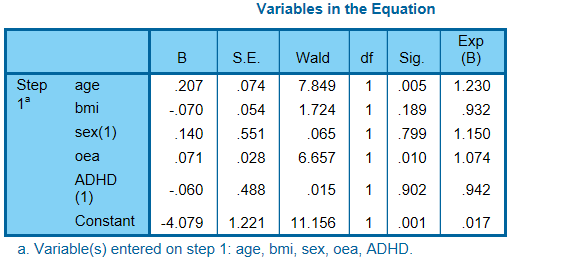


**PEA**


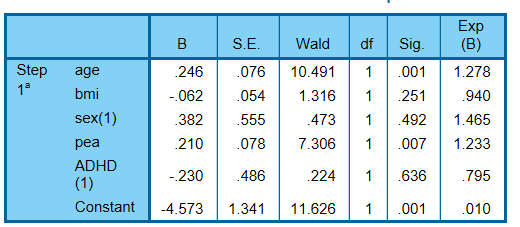

Supplement: Supplementary file 1 — Table S1. Serum levels of AEA, OEA, and PEA are independently associated with ASD status when adjusting for age, gender, BMI, and ADHD (logistic regression). (DOCX 64 kb) [file 13229_2019_256_MOESM1_ESM.docx]
